# Boosting *Purnica granatum* L. Seed Oil Yield: An Adaptive Neuro-Fuzzy Interference System Fuels SC-CO_2_ Extraction Breakthrough

**DOI:** 10.3390/foods13010161

**Published:** 2024-01-02

**Authors:** Padej Pao-la-or, Boonruang Marungsri, Pornariya Chirinang, Kakanang Posridee, Ratchadaporn Oonsivilai, Anant Oonsivilai

**Affiliations:** 1 School of Electrical Engineering, Institute of Engineering, Suranaree University of Technology, Nakhon Ratchasima 30000, Thailand; padej@sut.ac.th (P.P.-l.-o.); bmshvee@sut.ac.th (B.M.); 2 Applied Food and Nutrition Division, Faculty of Science and Technology, Phetchaburi Rajabhat University, Phetchaburi 76000, Thailand; pornariya.chi@mail.pbru.ac.th; 3 School of Food Technology, Institute of Agricultural Technology, Suranaree University of Technology, Nakhon Ratchasima 30000, Thailand; posridee.ka@gmail.com; 4 Health and Wellness Research Group, Suranaree University of Technology, Nakhon Ratchasima 30000, Thailand

**Keywords:** Box–Behnken design, percentage yield, oil, response surface methodology, adaptive neuro-fuzzy interference system, pomegranate seed

## Abstract

This study used supercritical fluid extraction to successfully enhance the conditions for extracting oil from pomegranate seeds. To determine the optimal extraction conditions for maximizing pomegranate oil yield, the researchers employed a Box–Behnken design experimental strategy, involving three parameters with three levels each: extraction pressure, extraction temperature, and extraction time. To determine the optimal optimization conditions, the Response Surface Method (RSM) and the Artificial Neural Fuzzy Intelligent System (ANFIS) were also used. The results revealed a strong correlation with the experimental data, demonstrating that both strategies were helpful in optimizing the extraction process. The ideal extraction parameters, according to this study, were an extraction pressure of 40 MPa, an extraction temperature of 55 °C, and an extraction time of 120 min with a CO_2_ flow rate of 21.3 L/h.

## 1. Introduction

Pomegranate seed oil obtained through cold extraction is classified as an aromatic essential oil. It is often used as an additive in cosmetics due to its rich oil content comprising antioxidant compounds and flavonoids. It is also popular in supplements because it is rich in fatty acids. Additionally, the important phytoestrogens found in pomegranate seed oil may also relieve the symptoms of menopause. Moreover, pomegranate oil may help to alleviate night sweats, prevent depression, and reduce vaginal dryness (although scientific data confirming this is lacking) [1]. According to Promprom [2], pomegranate seed and peel extracts are rich in phytoestrogens and exhibit estrogenic properties in ovariectomized rats. Pomegranate seed and peel extracts increase uterine wet weight and promote cornification of the vagina and proliferation of the uterine endometrium in ovariectomized rats. Pomegranate seed and peel extracts (at a dosage of 1000 mg/kg B.W.) tend to improve bone mineral densities, as determined by dual energy X-ray absorptiometry. Furthermore, several studies have found that pomegranate seed oil has a higher antioxidant capacity than red wine and is comparable to green tea extract [3].

Pomegranate products, including pomegranate seed oil (PSO) and fermented juice (PFJ), contain potent antioxidants and eicosanoid enzyme inhibitors in the form of flavonoids. Studies have shown that PSO flavonoids inhibit both COX-1 and LOX-1 activity, whereas PFJ flavonoids only inhibit LOX-1 activity. Furthermore, PSO has comparable antioxidant properties to butylated hydroxyanisole (BHA) and green tea. These findings suggest that pomegranate products may offer potential health benefits by safeguarding cells from oxidative damage and decreasing inflammation [4].

The process extracting oil from seeds involves complex stages that are time-consuming and typically result in a low yield. It is estimated that commercially marketed pomegranate seed oil, sold in one-pound increments, is derived from seeds weighing more than 200 pounds (91 kg). The oil can be stored in a cool and dry place for only 14 to 16 months. Therefore, the production of pomegranate oil is expensive. Currently, the sales price for commercial pomegranate seed oil is 15 baht per 220 cc. The countries that produce pomegranate seed oil include China, Egypt, India, and Turkey [1]. There are several methods to extract oil, such as solvent extraction, compression, etc. [5]. Supercritical CO_2_ extraction shines as a green champion in the extraction industry, offering safer and cleaner alternatives to traditional methods. Furthermore, this method delivers high-purity, consumer-friendly extracts. Its rise signals a promising shift towards sustainable and responsible extraction practices for the future [6].

Despite its growing popularity, the health benefits of pomegranate seed oil are still emerging. Although promising research suggests that its potent antioxidants, particularly punicic acid, may offer cardioprotective, anti-inflammatory, and blood-sugar-regulating effects, further studies are needed to solidify these claims and establish safe dosages. Although it shows promise as a complementary therapy for certain conditions, maintaining a balanced diet and a healthy lifestyle remain the pillars of good health. Pomegranate seed oil could serve as a promising addition to explore under the guidance of your healthcare provider [7].

Superheated hexane extraction (SHHE) is a promising technique for obtaining high-quality pomegranate seed oil with a high yield. The oil yield increases with higher temperatures and longer extraction times. The highest yield of 21.3% was attained under conditions of 70 °C for 30 min. The resulting oil possesses desirable characteristics, such as a light-yellow color, low acid value, high refractive index, and a rich content of bioactive compounds. These qualities make it suitable for various applications in the food, cosmetic, and pharmaceutical industries [8].

Pomegranate seed oil (PSO) boasts a wealth of bioactive compounds and potent antioxidant activity, making it a sought-after ingredient in food, cosmetics, and pharmaceuticals. Although PSO is traditionally extracted through cold pressing, this method faces challenges due to its low yield and high cost. This study investigated the SFE extraction of pomegranate seed oil (PSO) through a combination of experimental and modeling approaches [9,10,11]. Supercritical CO_2_ extraction (SC-CO_2_) has emerged as a superior alternative method, providing a high oil yield (17.1%), abundant bioactive compounds (with a punicic acid content of 60.6% and γ-tocopherol at 325.2 mg/100 g), and potent antioxidant activity comparable to reference antioxidants [12]. The optimal SC-CO_2_ parameters for PSO include a pressure of 35 MPa, a temperature of 60 °C, and an extraction time of 90 min. However, the longer extraction time may prompt the consideration of alternative methods, such as ultrasonic-assisted extraction (UAE) [13]. UAE delivers exceptional oil yields and quality, with a high bioactive content (punicic acid content of 65.3% and γ-tocopherol at 296.8 mg/100 g) and strong antioxidant activity, all achieved within a shorter 36-min timeframe. However, it necessitates higher energy consumption compared to other methods. Supercritical fluid extraction (SFE) also offers promise, with complex modeling approaches such as Sovová and Chrastil models enabling optimization and a high punicic acid content compared to traditional Soxhlet extraction [14]. Ultimately, the selection of the optimal extraction method depends on specific priorities. For achieving high yield, substantial bioactive content, and potent antioxidant activity, SC-CO_2_ and UAE reign supreme. For shorter processing times, SFE or other methods might be preferable. Considering the trade-offs between yield, quality, efficiency, and sustainability is crucial for selecting the most suitable PSO extraction technique.

Supercritical fluid extraction (SFE) offers immense potential for extracting bioactive compounds from seed oils; however, optimizing its complex parameters can be challenging. The Adaptive Neuro-Fuzzy Inference System (ANFIS) is a powerful tool that shines in this domain. Studies by Zhang et al. [15] and Li et al. [16] demonstrate its effectiveness, with ANFIS models achieving R² values exceeding 0.99 for predicting both yield and antioxidant activity/purity. These models identified optimal SFE conditions for specific goals, such as maximizing the yield (35 MPa pressure, 45 °C temperature) or purity (30 MPa pressure, 40 °C temperature). ANFIS’s strength lies in its ability to learn from data and adapt to the intricate nonlinear relationships between SFE parameters and the desired outcomes. This translates to improved extraction efficiency, resulting in higher quality oil with maximized bioactive content while minimizing impurities. The benefits extend beyond SFE, with ANFIS’s optimization capabilities finding applications in diverse fields, such as control systems, finance, and manufacturing. Overall, the ANFIS model emerges as a game-changer for optimizing SFE, paving the way for the more efficient, sustainable, and cost-effective extraction of valuable bioactive compounds from seed oils [17].

Seed oil extraction processes are being revolutionized by sophisticated optimization techniques, such as Response Surface Methodology (RSM) and the Adaptive Neuro-Fuzzy Inference System (ANFIS). Although both effectively model the intricate relationships between the extraction parameters and desired outcomes, ANFIS often shines with its superior prediction accuracy and optimization capabilities. Studies across diverse seed oils, including crude rubber, sea buckthorn, black cumin, and Nigella sativa, demonstrate this advantage. ANFIS models consistently achieve R² values that exceed 0.98 for yield prediction in SFE and successfully identify optimal conditions for maximizing yield and bioactive compound content [18,19,20,21,22]. Despite variations in specific pressure, temperature, and time values depending on the oil type, a pressure range of 30–35 MPa, a temperature range of 40–45 °C, and a 60-min extraction time have emerged as the optimal conditions for achieving high yield and quality across different studies. This trend underscores the potential of ANFIS to streamline and optimize seed oil extraction processes, leading to increased efficiency, improved product quality, and ultimately, a more sustainable and cost-effective approach for extracting valuable bioactive compounds from these diverse sources. Mathematical modeling methods, notably RSM, are crucial for evaluating and optimizing the extraction process. RSM combines mathematical and statistical principles to develop a mathematical model that describes the process, analyzes the impact of independent variables, and optimizes the processing parameters. This research investigates the elaborate relationship between the extraction parameters, namely pressure, temperature, and time, and their impact on pomegranate seed oil yield. By employing sophisticated optimization techniques (RSM and ANFIS), we aimed to uncover the ideal extraction conditions that maximize pomegranate seed oil yield.

## 2. Materials and Methods

### 2.1. Sample Preparation

Pomegranate seeds, sourced from Nakhon Ratchasima, Thailand, were subjected to a drying process within a tray dryer (Kluaynumtaitowop, Bangkok, Thailand) maintained at a temperature of 60 °C. Subsequently, the dried seeds were pulverized into a fine powder and stored under vacuum conditions at a temperature of 4 °C until further use.

### 2.2. Proximate Analysis

The proximate composition of the dried pomegranate seed powder was determined using standard analytical methods, including analyzing moisture (AOAC Method 925.10), ash (AOAC Method 900.02 A), protein (AOAC Method 928.08), fat (AOAC Method 945.16), crude fiber (AOAC Method 978.10), and carbohydrates (AOAC Method 995.13) [23].

### 2.3. Supercritical Carbondioxide Extraction (SC-CO_2_)

All SC-CO_2_ extraction experiments were conducted using a Spe-ed^®^ SFC supercritical fluid machine obtained from Applied Separations Inc., (Allentown, PA, USA). A precise amount of dried *Punica granatum* L. seed powder was carefully weighed and placed into the extraction chamber. Subsequently, the extracted PSO was weighed to determine the percentage yield of extraction using the formula below [5,6]:

Yield percentage = (oil (g)/seed powder (g)) × 100(1)

### 2.4. Experimental Design

The SC-CO_2_ conditions were optimized using Response Surface Methodology (RSM), a widely used optimization technique [24,25]. RSM was employed to determine the optimal levels of three extraction variables (pressure, temperature, and time), with respect to the extraction yield response (Table 1). The independent and dependent variables were as follows: extraction pressure (X_1_; 20, 30, and 40 MPa), extraction temperature (X_2_; 50, 55, and 60 °C), and extraction time (X_3_; 60, 90, and 120 min). The experiments were designed according to the Box–Behnken design, and the order of the experiments was completely randomized. The data were analyzed using regression analyses [5,6].

### 2.5. Experimental Validation of the Optimal Conditions

The extraction yield response was optimized by maximizing its value according to the developed model. To validate the model’s accuracy, three additional extraction experiments were conducted under the predicted optimal operating conditions, which differed from those used to develop the model.

### 2.6. ANFIS Modelling

ANFIS (Adaptive Neuro-Fuzzy Inference System) is a modeling technique that blends the strengths of fuzzy logic and neural networks to create a system capable of learning and making decisions based on input data. The ANFIS model comprises three crucial phases: fuzzification, inference engine, and defuzzification.

During the fuzzification phase, input values are converted into fuzzy sets using membership functions (MFs). Membership functions represent the degree to which a given input value belongs to each fuzzy set. This nonlinear mapping of inputs aids in capturing the linguistic information inherent in the data. Although various types of membership functions can be employed, the Gaussian shape was considered in this instance.

The inference engine phase involves constructing fuzzy rules to determine the output based on the fuzzy inputs. These rules typically follow an IF–THEN format, where the antecedent (IF) part outlines the conditions based on the fuzzy inputs, and the consequent (THEN) part dictates the output fuzzy set. The ANFIS model utilizes two methods to generate these rules: grid partitioning (GP) and subtractive clustering (SC). In this work, the SC method was adopted as it generates rules by clustering the data, resulting in a minimal number of rules.

Finally, the defuzzification phase transforms the fuzzy output into a crisp value that can be utilized for decision-making or further processing. Defuzzification methods determine the single output value based on the fuzzy sets and their respective degrees of membership. In this case, the weighted average method was employed.

Overall, the ANFIS model effectively combines fuzzy-logic-based fuzzy rules with the adaptive learning capabilities of neural networks. This powerful tool is well-suited for modeling complex systems and making decisions based on uncertain or imprecise data [18].

An example of the ANFIS rules is outlined below:

IF × is A_1_ and y is B_1_, then f 1 = g1(x, y)
IF × is A_2_ and y is B_2_, then f 2 = g2 (x, y)

Where, the As and Bs are the membership functions of the two inputs x and y. The output f is determined using the outputs of the two rules, f1 and f2 [18].

### 2.7. Fatty Acid Composition by GC Chromatography

Fatty acid methyl esters (FAMEs) were synthesized through a transmethylation reaction using boron trifluoride in methanol. The FAMEs were subsequently analyzed using an Agilent 7890 (Agilent, Santa Clara, CA, USA) a instrument equipped with a 100 m × 0.25 mm × 0.2 µm fused silica capillary column (SP2560, Supelco Inc., Bellefonte, PA, USA). A flame ionization detector (FID) was used, with both the injector and detector temperatures maintained at 260 °C. The column temperature was initially set at 70 °C and was then gradually increased at a rate of 13 °C/min until a temperature of 175 °C was reached. Following this, the temperature was further elevated at a rate of 4 °C/min until a temperature of 240 °C was reached. Helium was utilized as the carrier gas, flowing at a rate of 20 mL/min. A 1 µL aliquot of FAME was injected with a split ratio of 1:30. The identification and quantification of fatty acids were achieved by comparing the relative retention times of FAME peaks in the sample with those of standard compounds (37 component FAME Mix, catalog No. 47885-U, Supelco, Bellefonte, PA, USA) [6,26].

## 3. Results

### 3.1. Proximate Analysis

The pomegranate seed powder had a moisture content of 3.53%, an ash content of 1.56%, a fat content of 2.63%, a crude fiber content of 45.26%, and a protein content of 9.34%.

In comparing the proximate analysis results of dried pomegranate seed powder and dried winged bean seed powder, significant compositional differences were revealed (Table 2. Dried pomegranate seed powder had a lower moisture content (3.53%) than dried winged bean seed powder (9.22%), indicating more thorough drying of pomegranate seeds. Moreover, dried pomegranate seed powder had lower ash (1.56%) and fat (2.63%) levels compared to dried winged bean seed powder, which contained higher amounts of ash (4.91%) and fat (17.51%). However, dried winged bean seed powder had a higher protein content (33.83%) than dried pomegranate seed powder (9.34%). Additionally, dried winged bean seed powder contained significant amounts of crude fiber (12.23%) and carbohydrates (22.30%), whereas dried pomegranate seed powder had a considerably higher crude fiber content (45.26%) without specifying the carbohydrate content. These differences highlight the contrasting nutritional profiles of the two powders, suggesting their potential suitability for different applications in the food and nutraceutical industries [27].

### 3.2. Supercritical Carbondioxide Extraction (SC-CO_2_)

The optimization of pomegranate seed oil extraction was carried out using the same RSM methodology employed by Liu et al. [7]. However, the composition of the pomegranate seed oil in this study differed significantly from theirs, featuring a higher proportion of unsaturated fatty acids, particularly oleic acid, linoleic acid, and α-linolenic acid. It is confirm Table 3 summarizes the percentage yields (Y1) of pomegranate oil obtained from all the experiments, including the response surface method and ANFIS modeling.

The statistical analysis of the results was conducted using Analysis of Variance (ANOVA) to assess the significance of mean differences. Duncan’s procedure was utilized at a significance level of *p* ≤ 0.05 for the mean analysis. Additionally, Response Surface Methodology (RSM) was applied to determine the regression coefficients and to evaluate the statistical significance of the model terms. RSM enables the fitting of regression models to experimental data, facilitating the identification of an optimal region encompassing all studied response variables. Design-Expert^®^ software (Version 8.0.7.1, Stat-Ease, Inc., Minneapolis, MN, USA) was employed to meticulously craft the experimental design, rigorously analyze the collected data, and optimize the process parameters. Moreover, the data presented in Table 3 was subjected to One-Way ANOVA to establish correlations between the independent variables and to obtain the optimal extraction conditions for achieving the highest percentage yield, as outlined in Table 3.

According to Table 3, the optimal extraction conditions for pomegranate oil production were determined to be a pressure of 40 MPa, a temperature of 55 °C, and an extraction time of 120 min, resulting in a maximum yield of 4.50%. After placing the optimal condition value into the regression equation produced, the expected yield value was 4.55%. The results of the experiment revealed that extraction pressure affected the percentage yield of oil (*p* < 0.05) more than extraction temperature and extraction duration.

Furthermore, the data were tested to determine correlations between the independent variables and the yield %. The statistical analysis revealed a significant association (*p* < 0.01). Liu et al. [9] studied the extraction of pomegranate seed oil using SC-CO_2_ extraction. They found that the optimal extraction conditions were a pressure of 37.9 MPa, a temperature of 47.0 °C, and a CO_2_ flow rate of 21.3 L/h. Under these conditions, they were able to achieve an oil yield of 156.3 g/kg (dry basis) [9].

**Figure 1:**
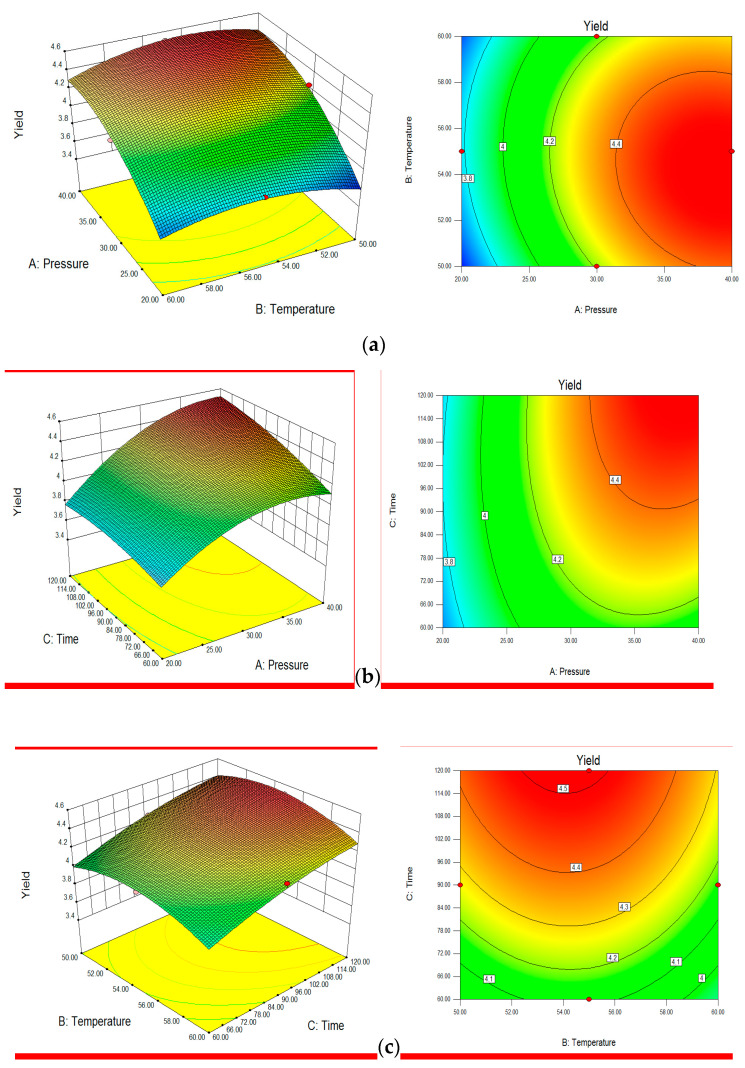
Response surface plots depicting the interactive effects of extraction pressure and time on the percentage yield of pomegranate oil (*Purnica granatum* L. oil) at a constant time of 120 min (**a**), a constant temperature of 55 °C (**b**), and a constant pressure of 40 MPa (**c**).

Table 4 provides an overview of the regression coefficients and the regression model. According to Raymond et al. [28], an R^2^ value of at least 0.80 indicates a good fit for a model. The high value of the coefficient of multiple determination (R^2^ = 0.9788) suggests that the model adequately represents the experimental results. The two methods of pomegranate seed oil extraction, SC-CO_2_ extraction and screw pressing, both have their own advantages and disadvantages. SC-CO_2_ extraction can achieve higher oil yields, but it is more expensive and requires specialized equipment. Screw pressing is simpler and less expensive, but it may not be as efficient at extracting oil. The choice of extraction method depends on the specific needs of the producer. If a high oil yield is required, then SC-CO_2_ extraction may be the best choice. However, if cost is a major concern, then screw pressing may be a more viable option. Response surfaces can be visualized through three-dimensional plots, illustrating the functional response of two factors while keeping the other factor constant. The relationship between the extraction pressure, extraction temperature, and extraction time and the highest yield of pomegranate seed oil is depicted in Figure 1.

### 3.3. Fitting Model

The regression coefficients were derived from the percentage yields of pomegranate oil obtained in each experiment. The resulting model was statistically significant, indicating that at least one of the extraction variables was responsible for the variation in the response variable. The regression coefficients and the regression model are presented in the equation for *Purnita granatum* L. seed oil.

Y1 = −23.03375 + 0.189X_1_ + 0.8475X_2_ + 0.015583X_3_ − 8.5 * 10^−4^ X_1_ X_2_ + 2.75 * 10^−4^ X_1_ X_3_ – 5 * 10^−5^ X_2_ X_3_ − 2.313 * 10^−3^ X_12_ − 7.45 * 10^−3^ X_22_ − 9.6 * 10^−5^ X_32_ (2)

The model showed a strong correlation with the experimental data, as evidenced by the high R-squared value of 0.9788. Additionally, the *p*-value obtained from the lack-of-fit test confirmed the model’s ability to accurately represent the experimental findings. These results collectively demonstrate the model’s effectiveness in capturing and representing the observed experimental data.

### 3.4. Analysis of Regression Coefficients

Increasing the extraction pressure considerably increased the oil extraction yield (*p* < 0.0001). Increasing the extraction time also tended to increase the yield (*p* < 0.05). However, after a certain point, further increasing the temperature reduced the yield, indicating an ideal temperature range (Figure 1) Reaction surfaces can be shown using three-dimensional plots that depict the reaction as a function of two factors while holding the third constant. The winged bean seed oil yield was strongly impacted by extraction pressure, temperature, and time (Figure 1). The response surface plot (Figure 1) reveals the interactive influence of extraction time and temperature on pomegranate seed oil yield when maintaining a constant extraction pressure of 40 MPa. The plot effectively demonstrates the synergistic relationship between these parameters in optimizing oil yield.

### 3.5. Validation of the Model

The most effective extraction method for pomegranate seed oil was determined by combining the extraction factors that resulted in the highest yield (22.29% and 4.50%). These conditions included an extraction pressure of 40 MPa, extraction temperatures of 50 °C and 55 °C, and extraction times of 90 and 120 min. Three extractions were performed under these conditions to verify the model’s predictive capacity (Table 4). The predicted and experimental values of the percentage yield were in agreement, confirming the accuracy of the model. Furthermore, the data were analyzed to determine the correlation between independent variables and the percentage yield. The statistical analysis was significant, with a *p*-value less than 0.01.

### 3.6. Data Acquisition and Preprocessing for ANFIS

**Figure 2:**
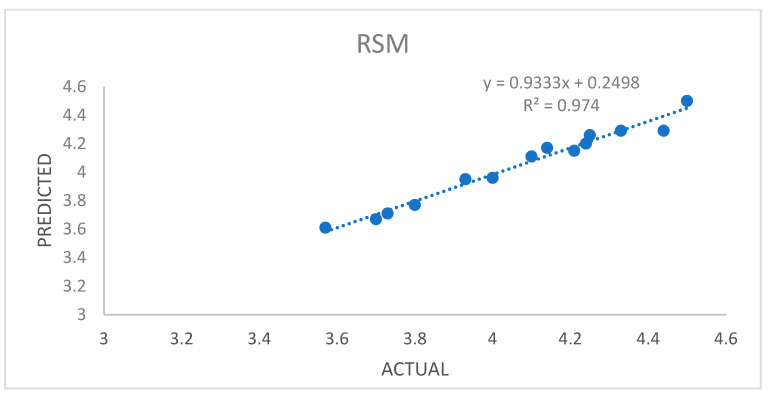
A plot of predicted and experimental values for the % yield of oil extracted from pomegranate seeds using RSM.

**Figure 3:**
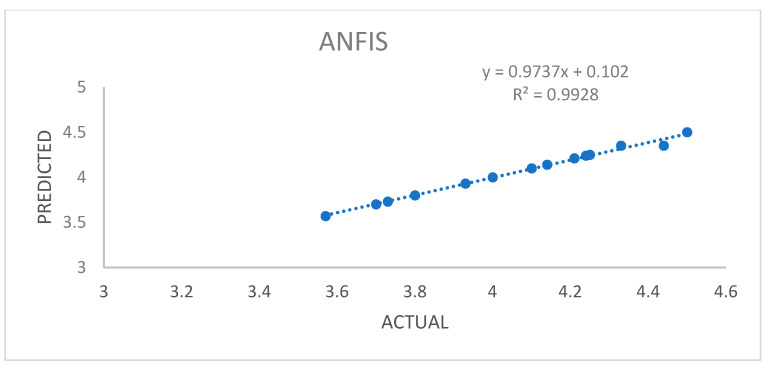
A plot of predicted and experimental values for the % yield of oil extracted from pomegranate seeds using the ANFIS model.

In the laboratory setting, determining the optimal extraction conditions can be a lengthy and complicated process, especially when dealing with three variables, each with three levels. To simplify this process and reduce the number of experiments, the Box–Behnken design is often employed. However, for achieving even better optimization, the Adaptive Neuro-Fuzzy Inference System (ANFIS) has proven to be a more effective approach compared to Response Surface Methodology (RSM). The Adaptive Neuro-Fuzzy Inference System (ANFIS) stands out as a valuable tool for optimization problems due to its ability to effectively combine the strengths of neural networks and fuzzy logic. The ANFIS model is particularly adept at handling intricate and nonlinear relationships between variables, making it a powerful instrument for optimizing various systems and processes. The performance of the ANFIS model is evaluated by comparing actual and predicted values and visually representing the linear regression analysis. Furthermore, as demonstrated in Figure 2 and Figure 3, the ANFIS model offers valuable insights into its predictive accuracy compared to RSM.

### 3.7. Fatty Acid Composition using GC Chromatography

**Figure 4:**
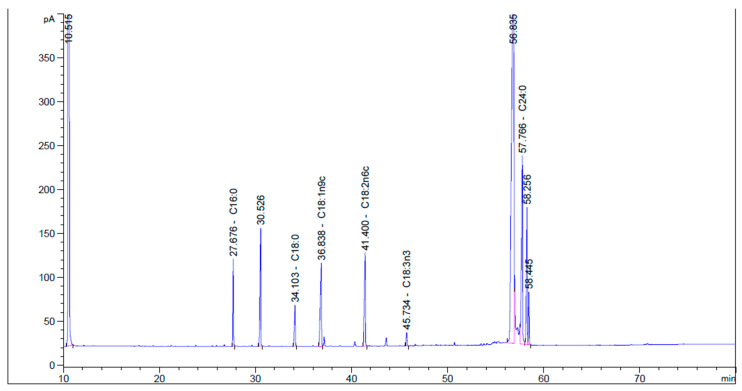
Gas chromatography of fatty acids from pomegranate seed oil using the SC-CO_2_ extraction method.

The analysis of pomegranate seed oil using gas chromatography (GC) revealed the presence of various fatty acids, including saturated and unsaturated varieties. The saturated fatty acids identified in pomegranate seed oil include palmitic acid (C16:0), stearic acid (C18:0), and lignoceric acid (C24:0). Additionally, pomegranate seed oil contains unsaturated fatty acids, such as oleic acid (C18:1n9c), linoleic acid (C18:2n6c), and α-linoleic acid (C18:3n3). A detailed breakdown of the fatty acid profile of pomegranate seed oil is presented in Table 5 and Figure 4.

## 4. Discussion

When comparing the models, both Kumar et al. [29] and Jisieike et al. [18] found the RSM and ANFIS models effective in predicting biodiesel yield with high R^2^ values (>0.97). Jisieike et al. [18] further compared optimization algorithms, with ANFIS-PSO achieving the lowest final FFA content. These results suggest that both models are valuable tools, with ANFIS potentially offering superior optimization capability.

Abbasi et al. [30] compared several extraction methods and found that SC-CO_2_ using hexane as the modifier provided the highest yield. However, they highlighted significant differences in the fatty acid profiles of the oil depending on the method used. Their results are not directly comparable to the seed oil profile presented in this study due to the use of an unknown extraction method.

The fatty acid profile of the seed oil provided in this research differs significantly from Abbasi et al.’s SC-CO_2_ extraction with hexane in several key fatty acids, particularly palmitic, stearic, oleic, α-linolenic, and lignoceric acids. This implies that the extraction method used significantly impacts the fatty acid composition and potentially other oil properties.

Optimizing biodiesel production and maximizing the quality of pomegranate seed oil both demand strategic choices, including the selection of appropriate models for precise prediction and extraction methods that yield the desired composition. Although RSM excels in robust modeling, ANFIS, particularly when paired with PSO, holds promise for superior optimization in biodiesel production. Further research comparing their performance across diverse feedstocks and catalysts is crucial. For pomegranate seed oil, the stark differences in fatty acid profiles highlight the significant influence of the extraction methods on the properties of the oil. Future studies should investigate how method choice not only affects quality, but also the antioxidant content and potential health benefits. Ultimately, both biodiesel and pomegranate seed oil applications must consider cost-effectiveness and scalability for successful industrial implementation. This necessitates the careful consideration of both optimization strategies and extraction methods, ensuring a balance between the desired outcomes and economic feasibility.

## 5. Conclusions

The optimal conditions for extracting pomegranate seed oil with the highest percentage yield were determined using a randomized Box–Behnken design. The data were statistically analyzed using multiple regression. The conditions that yielded the highest percentage yield were an extraction pressure of 40 MPa, an extraction temperature of 55 °C, and an extraction time of 120 min, which resulted in a yield of 4.50%. Future research should focus on determining the fatty acid profile and bioactive compounds of pomegranate seed oil.

## Figures and Tables

**Table 1:** Encoded and coded levels of independent variables used in the experimental design.

Symbols	Independent Variables	Coded Levels		
		−1	0	1
X_1_	Extraction pressure (MPa)	20	30	40
X_2_	Extraction temperature (°C)	50	55	60
X_3_	Extraction time (min)	60	90	120

**Table 2:** Proximate analysis.

Components	Content (%)
Moisture	3.53 ± 0.15
Ash	1.56 ± 0.09
Protein	9.34 ± 0.38
Fat	2.63 ± 0.24
Crude fiber	45.26 ± 0.76
Carbohydrate	37.68 ± 0.70

**Table 3:** Experimental design and response of independent variables to the extract parameters.

Exp. No.^a^	Independent Variables			*Punica granatum* L.		
	Pressure (MPa) X_1_	Temp. (°C) X_2_	Time (min) X_3_	% Yield (Y_1_) Experimental	% Yield (Y_1_) RSM-Predicted	% Yield (Y_1_) ANFIS-Predicted
1	20	50	90	3.57 ± 0.38 ^d^	3.57	3.57
2	20	55	60	3.73 ± 0.28 ^bcd^	3.73	3.73
3	20	55	120	3.80 ± 0.18 ^bcd^	3.8	3.80
4	20	60	90	3.70 ± 0.12 ^cd^	3.70	3.70
5	30	50	60	4.00 ± 0.21 ^abcd^	4.00	4.00
6	30	50	120	4.24 ± 0.31 ^abc^	4.24	4.24
7	30	55	90	4.33 ± 0.10 ^ab^	4.35	4.35
8	30	55	90	4.44 ± 0.06 ^a^	4.35	4.35
9	30	55	90	4.28 ± 0.22 ^abc^	3.93	3.93
10	30	60	60	3.93 ± 0.29 ^abcd^	4.14	4.14
11	30	60	120	4.14 ± 0.04 ^abc^	4.25	4.25
12	40	50	90	4.25 ± 0.64 ^abc^	4.10	4.10
13	40	55	60	4.10 ± 0.62 ^abcd^	4.50	4.50
14	40	55	120	4.50 ± 0.13 ^a^	4.21	4.21
15	40	60	90	4.21 ± 0.36 ^abc^	3.57	3.57

^a^ Experiments were conducted in a random order. ^b,c,d^ Same letter show no significant difference at *p* < 0.05, different letter show significant difference (*p* < 0.05) in the same column.

**Table 4:** Analysis of variance (ANOVA) of independent variables for the extraction of oil from pomegranate seeds. R-squared = 0.9788. df, degree of freedom.

Source	*Purnica granatum* L.					
	Sum of Squares	df	Mean Square	F Value	*p*-Value Prob > F	Significant
Model	1.09	9	0.1216	25.66	0.0012	significant
A-pressure	0.6384	1	0.6384	134.69	<0.0001	
B-temp	0.0008	1	0.0008	0.1688	0.6982	
C-time	0.1058	1	0.1058	22.32	0.0052	
AB	0.0072	1	0.0072	1.52	0.2718	
AC	0.0272	1	0.0272	5.74	0.0619	
BC	0.0002	1	0.0002	0.0475	0.8361	
A^2^	0.1975	1	0.1975	41.66	0.0013	
B^2^	0.1281	1	0.1281	27.02	0.0035	
C^2^	0.0275	1	0.0275	5.79	0.0611	
Residual	0.0237	5	0.0047			
Lack of Fit	0.0103	3	0.0034	0.5124	0.7135	not significant
Pure Error	0.0134	2	0.0067			
Cor Total	1.12	14				
Std.Dev = 0.0688 R-Squared = 0.9788 Mean = 4.08 R-Squared = 0.9407 C.V.% = 1.69 Adeq Precision = 15.9213						
	
	

**Table 5:** Fatty acid profile of *Purnica granatum* L. oil prepared by SC-CO_2_ extraction.

Ret Time (min)	Type	Area (Pa·s)	Amt/Area	Norm (%)	Grp	Name
27.676	BB	427.21164	9.55544 × 10^−4^	9.990683	Palmitic acid sat	C16:0
34.103	BB	323.84680	9.45592 × 10^−4^	7.494542	Stearic acid sat	C18:0
36.838	BV	837.20972	9.41893 × 10^−4^	19.299116	Oleic acid w-9 FA	C18:1n9c
41.400	BB	796.17188	9.88680 × 10^−4^	19.264782	Linoleic acid	C18:2n6c
45.734	BB	96.01950	1.23414 × 10^−3^	2.900177	α-Linolenic acid ALA	C18:3n3
57.766	VB	1796.60498	9.33612 × 10^−4^	41.050699	Lignoceric acid	C24:0
Total				100.000000		

## Data Availability

The data are contained within the article.
